# Needs assessment and planning for a clinic-community-based implementation program for hypertension control among blacks in New York City: a protocol paper

**DOI:** 10.1186/s43058-022-00340-z

**Published:** 2022-09-06

**Authors:** Joyce Gyamfi, Claire Cooper, Aigna Barber, Deborah Onakomaiya, Wen-Yu Lee, Jennifer Zanowiak, Moses Mansu, Laura Diaz, Linda Thompson, Roger Abrams, Antoinette Schoenthaler, Nadia Islam, Gbenga Ogedegbe

**Affiliations:** 1 New York University School of Global Public Health, 708 Broadway, New York, NY 10003 USA; 2 Department of Population Health, New York University School of Medicine, New York, NY USA; 3 Vilcek Institute of Graduate Biomedical Sciences, New York University Grossman School of Medicine, New York, NY USA; 4 NYU Grossman School of Medicine, NYU Langone Health, 180 Madison Avenue, New York, NY 10016 USA; 5 Institute for Excellence in Health Equity, New York University Langone Health, New York, NY USA; 6 Center for Healthful Behavior Change, Institute for Excellence in Health Equity, NYU Langone Health, New York, NY USA

**Keywords:** Hypertension, Blood pressure control, Blacks, Needs assessment, Implementation context, Clinic-community-based partnerships

## Abstract

**Background:**

Hypertension (HTN) control among Blacks in the USA has become a major public health challenge. Barriers to HTN control exist at multiple levels including patient, physician, and the health system. Patients also encounter significant community-level barriers, such as poor linkage to social services that impact health (unstable housing, food access, transportation). We describe a multi-component needs assessment to inform the development, implementation, and evaluation of a program to improve HTN management within a large healthcare system in New York City (NYC).

**Methods:**

Guided by the Community-Based Participatory Research (CBPR) and Consolidated Framework for Implementation Research (CFIR) frameworks, data will be collected from four main sources: (1) quantitative surveys with health systems leadership, providers, and staff and with community-based organizations (CBOs) and faith-based organizations (FBOs); (2) qualitative interviews and focus groups with health systems leadership, providers, and staff and with CBOs and FBOs; (3) NYC Community Health Survey (CHS); and (4) New York University (NYU) Health system Epic Electronic Health Record (EHR) system. The data sources will allow for triangulation and synthesis of findings.

**Discussion:**

Findings from this comprehensive needs assessment will inform the development of a clinic-community-based practice facilitation program utilizing three multi-level evidence-based interventions (nurse case management, remote blood pressure (BP) monitoring, and social determinants of health (SDoH) support) integrated as a community-clinic linkage model for improved HTN control in Black patients. Integration of stakeholders’ priorities, perspectives, and practices into the development of the program will improve adoption, sustainability, and the potential for scale-up.

**Trial registration:**

NCT05208450; registered on January 26, 2022

Contributions to the literature

- Community-clinic linkage models (CCLMs) provide an opportunity to unite stakeholders to implement comprehensive evidence-based interventions (EBIs) into real-world settings, targeting underserved populations, to mitigate existing disparities.
- To optimize adoption and sustainability of the model, the implementation context must be assessed to understand barriers and facilitators to EBI implementation.
- This protocol synthesizes a comprehensive range of quantitative and qualitative strategies including diverse stakeholder feedback, characterization of the hypertensive population, and assessment of implementation context within primary care clinics and the community to inform the development of a CCLM and PF strategy tailored to the target population and context.

## Background

Hypertension (HTN) remains a major public health challenge in the USA. Nearly half (45%) of North American adults have been diagnosed with HTN [1], and of those, only about half (53%) have their HTN under control [2]. Uncontrolled HTN is a leading cause of cardiovascular-related deaths and drives high healthcare expenditure nationally [3]. As a precursor to many cardiovascular outcomes, it also serves as the single most influential driver of the mortality gap between Blacks and Whites [4]. HTN diagnoses are disproportionately high among non-White racial/ethnic groups, with rates in the USA highest among Blacks [5]. This disparity is particularly stark in New York City (NYC), where 43.5% of Blacks are diagnosed with HTN vs 27.5% of Whites [3], and is underscored by the low HTN control rates that Blacks exhibit nationally (48.5%) when compared with Whites (55.7%) [2].

Achieving HTN control is essential for improving cardiovascular outcomes and reducing disparities. However, barriers to HTN control exist at multiple levels, including the patient, physician, health system, and community levels. At the patient level, <50% of individuals are estimated to adhere to their prescribed antihypertensive medications after 1 year of treatment [4], even though high adherence is a requisite for HTN control [5]. This trend is most prevalent among African Americans [6–9]. Lack of patient engagement also represents a significant barrier to blood pressure (BP) control, despite the evidence that higher patient engagement leads to a greater reduction in BP [10]. At the physician level, clinical inertia contributes to uncontrolled HTN: estimates of medication initiation by primary care physicians have been as low as 26%, and intensification is 16%, for patients diagnosed with HTN [11]. Poor integration of clinical decision support (CDS) tools into care can serve as a barrier at the health system level [12], whereas poor linkage to social services resources, which can influence health outcomes, impedes HTN control at the community level [12, 13].

The co-existence of numerous multi-level barriers to HTN control underscores both the challenge and the importance of developing efficacious interventions that improve HTN outcomes and address disparities. Several multi-level evidence-based interventions (EBIs) exist to address HTN control including remote BP monitoring (RBPM) [14–19], and incorporation of nurse case management (NCM) and community health workers (CHWs) into team-based care [20]. Numerous trials have recorded decreases in BP associated with use of RBPM [19, 21–24]. This effect may be even greater among Blacks, as illustrated by a randomized control trial conducted by Roger and colleagues which showed a two times greater reduction among Black RBPM users than among White participants [19]. Integration of NCM and CHWs into team-based care is also effective for improving HTN control. Both have shown positive impact on patient- and health system-level barriers (e.g., can improve medication adherence), resulting in demonstrated HTN improvement among patients [20]. CHWs are effective at engaging community members in evidence-based BP management strategies and addressing social determinants of health (SDoH) needs [25–27], bridging the gap between healthcare systems and underserved communities, particularly as part of a team-based care unit in primary care settings [28, 29]. Prior evidence demonstrates the effectiveness of using CHWs to help minority patients achieve improved HTN outcomes [30, 31].

Despite its effectiveness, adoption of EBIs in primary care settings for HTN control among Blacks is suboptimal and requires leveraging multiple-level partnerships to improve reach and sustainability. To achieve population health goals and health equity, key stakeholders such as providers, insurance companies, and community-based organizations (CBOs) must collaborate and sustain cooperation to encourage EBI adoption among Black community members for improved outcomes [32].

Community-clinic linkage models (CCLMs) represent an opportunity to bring together key stakeholders to enhance the effectiveness and adoption of EBIs in real-world settings. Islam and colleagues define CCLMs as “partnerships to help connect health care providers, community organizations, and public health agencies so that they can improve patients' access to preventive care, chronic care, and social services” [33]. They are often composed of multiple EBIs, integrated into a comprehensive intervention which targets complex health problems at multiple levels of influence. Despite the inherent benefits of CCLMs, healthcare contexts present unique challenges to their implementation, due to logistical (financial, administrative), patient (reimbursement challenges), and practice (challenges integrating interventions into workflows) factors [34, 35]. Furthermore, interventions that target the Black community must be adapted to address unique cultural contexts. Coupled with the inordinate time often needed to translate EBIs into real world settings (up to 17 years) [36], these factors highlight the need for an implementation strategy to facilitate an effective and timely translation process. Practice facilitation (PF) is one such implementation model. Used for tailoring and scaling interventions to healthcare settings, PF endeavors to build organizational capacity to support the integration of such EBIs into existing practice workflows [37, 38], whilst engaging key stakeholders in the healthcare system and the community.

The objective of this paper is to describe a multi-component, multi-level mixed methods needs assessment for a HTN management program guided by the Community-Based Participatory Research (CBPR) [39–43] and Consolidated Framework for Implementation Research (CFIR) [44] frameworks. The needs assessment will evaluate the context, barriers, and facilitators of implementing a CCLM comprised of three EBIs (NCM, RBPM, and SDoH support using a PF strategy; collectively termed *Practice Support and Community Engagement* [PACE]) within primary care practices in New York City. The needs assessment results will be applied to inform the development of the tailored PF strategy.

## Methods

### Study design

#### Brief overview of the needs assessment for the clinic-community-based implementation program for hypertension control

**Fig. 1:**
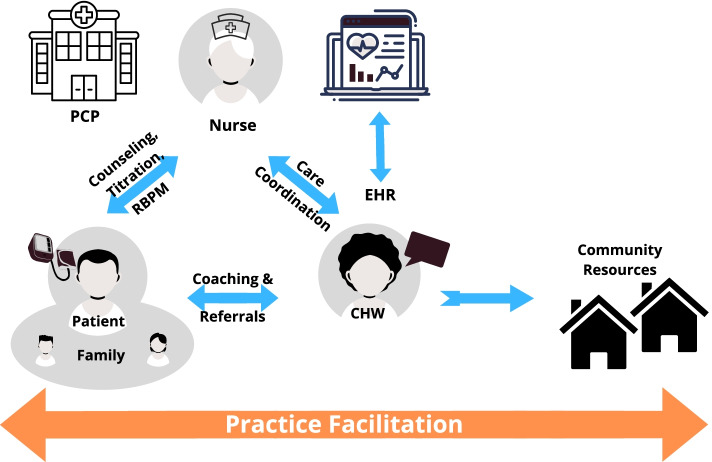
PACE intervention framework

The needs assessment for the program will be carried out between September 2020 and August 2023. The program will target Blacks with uncontrolled HTN followed in 27 primary care practices within the New York University Langone Health (NYULH) system. The program consists of two phases: (1) the pre-implementation phase, which comprises the needs assessment and refinement of the PF strategy, and (2) the implementation phase, which implements and evaluates a community-clinic linkage model. This model comprises three EBIs collectively termed PACE, which includes HBPM, NCM, and SDoH Support. Practice facilitators will assist each of the 25 primary care practices plus 2 pilot sites in the implementation of PACE (Fig. 1), in a step-wedged cluster randomized control trial design [45–48].

To maximize the effectiveness of the program on HTN-related disparities, PACE must be implemented and sustained within the primary care settings where Black communities receive care. Thus, the aim of this needs assessment study is to assess the varying contexts in which PACE will be implemented, in order to develop a PF strategy that is optimized for adoption, fidelity, and sustainment of the program. To achieve this aim, we will use a mixed-methods (quantitative and qualitative) approach to carry out a series of activities to tailor and refine the PACE intervention (see the “Primary data” and “Secondary data” sections for additional details). Results will guide the selection of a PF strategy that is tailored to address the barriers and facilitators identified.

### Conceptual frameworks

Two conceptual frameworks will guide this needs assessment: the Community-Based Participatory Research framework and the Consolidated Framework for Implementation Research. The CBPR framework will guide the overall conduct of the study. CBPR is a community engagement research framework that elevates the role of community partners and stakeholders in study design, implementation, evaluation, and dissemination [39–43]. CBPR centers community partner and stakeholder experience during the research process, acknowledging the stakeholders’ invaluable insight, whilst emphasizing equal partnership throughout all stages of the research process [39–43]. A community-engaged approach is crucial for optimizing implementation, and this needs assessment study will be conducted in partnership with an established Community Advisory Board (CAB) through all phases of the research.

The CFIR framework [44] will guide the implementation of the study activities, as well as interpretation of the data. CFIR comprises 5 domains that characterize the implementation context for a given intervention: (1) inner setting (e.g., leadership support, practice capacity); (2) outer setting (e.g., patient needs, external resources, and incentives); (3) individual characteristics (e.g., self-efficacy for counseling, conducting community referrals); (4) intervention characteristics (e.g., the complexity of PACE); and (5) process. CFIR will be used to identify the barriers and facilitators that exist across different levels within NYULH (i.e., patient, physician, health system, and community), yielding a comprehensive, formative evaluation of the PACE implementation context.

We describe the primary and secondary data sources and analyses plan below. Data will be collected from four main sources: (1) Quantitative surveys conducted with health systems leadership, providers, and staff and with CBOs and faith-based organizations (FBOs); (2) Qualitative interviews and focus groups with health systems leadership, providers, and staff and with community and faith-based organizations; (3) New York City Community Health Survey; and (4) NYU Langone Health (NYULH) system Epic Electronic Health Record (EHR). The data sources will allow for triangulation and synthesis of findings.

### Primary data

#### Study settings and participants

##### Study settings

To assess context at multiple levels, the study activities will be carried out in a number of settings. The surveys, interviews, and focus groups will be implemented in primary care practices affiliated with NYULH (inner setting) and participating CBOs and FBOs (outer setting). Primary care practices identified as implementation sites are located in Brooklyn, Manhattan, Queens, and Greater Long Island. They contain a range of approximately 70–2400 eligible patients (i.e., Black patients with a HTN diagnosis and at least 1 uncontrolled BP reading over a 2-year period between 2018–2021).

##### Participants

This study targets health system employees (primary care site providers, leadership, and staff; organizational leadership; Nurses and CHW/health navigators) and members and leadership of CBOs and FBOs serving the Black community. Individuals will be eligible to participate in the needs assessment if they (1) represent one of the stated personnel roles at NYULH or participating CBO/FBOs and (2) are able and willing to provide consent. Individuals who refuse to participate will be excluded from the study.

#### Recruitment strategies

##### Primary care practices

Using EHR data, the study team will identify 2 primary care practices for the pilot and 25 additional sites who serve at least 40% of hypertensive Black patients to participate in the intervention for the main trial. In consultation with the health system and practice site leadership, the study team will invite and enroll eligible and interested practices through email communications and scheduled in-person meetings at the sites. Practices are eligible to participate if (1) the practice is affiliated with NYULH and (2) the practice has used the Epic EHR for at least 12 months.

##### Health system clinical/non-clinical staff and sample size

Eligible health system clinical and non-clinical staff, including organizational leadership, practice site providers and staff, nurse case managers, and CHWs, will be identified and recruited in collaboration with health system leadership and other key individuals. Study team members will use a variety of recruitment strategies, including attending staff meetings at the practices, email outreach, and scheduling additional in-person meetings at the practices. Approximately 1–2 staff members from each of the 27 participating practice sites (includes 2 pilot sites), 10 organizational leaders, and 8 NCM/CHWs will be recruited to complete the surveys and interviews or focus groups.

##### CBO/FBO leadership/members and sample size

In collaboration with community partners and study team CHWs, we will identify eligible CBOs and FBOs that serve the social and economic needs of the Black community within the study catchment area. Study team members will attend community meetings at potential CBO/FBO partner sites and will send email communications to recruit participants. Approximately 1–2 members and leaders from each of the CBO/FBO sites will be recruited to participate in the surveys and interviews.

#### Data collection

Primary data for the needs assessment will be collected from surveys, interviews, and focus groups conducted among NYULH leadership, providers, and staff, as well as CBOs and FBOs.

All primary survey data collected from clinical and non-clinical staff both at NYULH and at CBOs and FBOs will be administered and collected through a Health Insurance Portability and Accountability Act (HIPAA) compliant web-based data collection tool (i.e., Research Electronic Data Capture [REDCap]). All data collected in REDCap will be stored in a secure, online database. All interviews and focus groups will be conducted by a member of the study team, trained in qualitative methods using a semi-structured moderator’s guide. All qualitative data collected from study respondents will be recorded either on audio recording devices (in-person sessions) or via the secure WebEx platform (virtual sessions) and will be transcribed verbatim by study staff.

### Measures

#### Surveys among health system leadership, providers, and staff and with CBOs and FBOs

##### Survey measures

Quantitative data will also be collected via various survey measures using the HIPAA-compliant REDCap web-based tool. Survey forms across all personnel types (i.e., NYULH leadership, practice site leadership, providers, staff, NCM/CHWs, and CBO/FBOs) will begin with a demographic and site characteristic questionnaire which includes information on participant demographics, experiences with a HTN management program, and characteristics about the organization.

Institutional and practice site personnel will receive measures in addition to the demographic form as follows (see Table 1 for scale descriptions). NYULH Leadership (e.g., Director of Nursing for NYULH) will receive (1) The Implementation Climate Scale (Shortened version); (2) The Stress/Discrimination in Hypertension Management Scale; and (3) The Scalability Assessment. Practice Site Leadership (e.g., Medical Director) will receive 4 survey measures in addition to the demographic form: (1) The Implementation Leadership Scale; (2) The Perception of PACE scale (guided by CFIR); (3) The Scalability Assessment; and (4) The Stress/Discrimination in Hypertension Management Scale. Practice Site Providers (e.g., Physicians, Nurse Practitioners) will receive 5 survey measures: (1) The Implementation Climate Scale; (2) The Provider Needs Assessment Scale; (3) The Implementation Leadership Scale (provider version); (4) Perceptions of PACE Scale; and (5) The Stress/Discrimination in Hypertension Management Scale. Practice Site Staff (e.g., Medical Assistants, NCMs, CHWs) will receive 4 survey measures: (1) Implementation Leadership Scale (staff version); (2) The Practice Needs Assessment; (3) Perceptions of PACE Scale; and (4) Stress/Discrimination in Hypertension Management Scale. CBOs/FBOs will not receive any forms except for the demographic/site characteristics form.

**Table 1:** Needs assessment scales administered to institutional and practice site personnel

Scale	Description	Personnel type administered scale			
		Institutional (NYULH) Leadership *(e.g., Director of Nursing for NYULH)*	Practice Site Leadership *(e.g., Medical Director)*	Practice Site Providers *(e.g., Physicians, Nurse Practitioners)*	Practice Site and Institutional Staff *(e.g., Medical Assistants, NCMs, CHWs)*
Implementation Climate Scale	Assess organizational context for EBI implementation	X (shortened version)		X	
Stress/Discrimination in Hypertension Management Scale	Assess perceptions of stress and racial discrimination experienced by patients at a healthcare organization	X	X	X	X
Implementation Leadership Scale	Examines the extent to which a leader is proactive, supportive, perseverant, and knowledgeable towards implementation, and the degree to which a hypertension management and control program can be implemented		X	X (provider version)	X (staff version)
Perceptions of PACE scale (CFIR-guided)	Guided by CFIR, this scale examines the practice culture, leadership attitudes, and beliefs around implementing PACE		X	X	X
Scalability Assessment	Assess the degree to which the PACE intervention is scalable	X	X		
Provider Needs Assessment Scale	Examines provider-specific context and needs regarding implementation of a HTN management program			X	
Practice Needs Assessment Scale	Examines practice site-specific context and needs regarding implementation of a HTN management program				X

#### Interviews and focus groups with health systems leadership, providers, staff and with CBOs and FBOs

##### Interview and focus group measures

Three types of qualitative instruments will be developed for the needs assessment, which will be targeted toward NCM/CHWs, CBO/FBOs, and NYULH Leaders/Providers. Table 2 provides an overview of the qualitative guides.

**Table 2:** Overview of needs assessment qualitative guides

Qualitative guide	Description	Domains	Constructs
CFIR Qualitative Focus Group Guide for NCMs and CHWs	Explore NCM and CHW perspectives of potential barriers and facilitators for the adoption of a hypertension management program at NYULH practice sites	Inner Setting	Readiness for implementation (NCM/CHWs, leadership), implementation climate (compatibility, relative priority, organizational incentives, and rewards), structural characteristics, tension for change, networks and communications, implementation climate (learning climate, goals, and feedback), and organizational culture.
		Outer Setting	Peer pressure, patient needs, and patient resources
		Individual Characteristics	Knowledge and beliefs about the intervention, self-efficacy, individual stage of change, personal attributes (i.e., skills for implementing the program), and individual identification with the organization
		Intervention Characteristics	Relative advantage, trialability, complexity, and design quality and packaging
Qualitative Interview Guide for CBOs and FBOs	Assess capacity and readiness for developing and integrating a community-clinic referral linkage program at CBOs and FBOs	Background/Organizational Structure	Organizational characteristics and existing organizational structures
		Readiness and Capacity	Organizational preparedness, organizational capacity
		Organizational Workflow	Referral and partnership workflows
		Partnership Evaluation	Organizational attitudes toward partnership formation with outside organizations, organizational beliefs about partnership formation with outside organizations
		Experiences of Stress, Racism, and Racial Discrimination	Perceptions of community-level stress, perceptions of community-level racism, perceptions of community-level racial discrimination
CFIR Qualitative Interview Guide for Providers, Practice Site Leaders, and NYULH Institutional Leaders	Explore Provider, Practice Site Leadership, and Institutional Leadership perspectives of potential barriers and facilitators for the adoption of a hypertension management program within NYULH	Inner Setting	Organizational capacity to implement hypertension management programs; organizational capacity to administer hypertension management programs
		Outer Setting	Perceptions of patient experiences with a hypertension management program
		Engagement	Leadership engagement with Nurses working on hypertension management
		Stress/Discrimination in Hypertension Management	Perceived organizational-level experiences of racism/discrimination, perceived organizational-level experience of stress

The NCM/CHW instruments (Table 2) are guided by CFIR and will explore the inner setting, outer setting, and characteristics of both the individual and the intervention. Inner Setting questions will capture readiness for implementation (NCM/CHWs, leadership), implementation climate (compatibility, relative priority, organizational incentives, and rewards), structural characteristics, tension for change, networks and communications, implementation climate (learning climate, goals, and feedback), and organizational culture. Outer Setting questions will include peer pressure, patient needs, and patient resources. The “Characteristics of Individual” questions will capture knowledge and beliefs about the intervention, self-efficacy, individual stage of change, personal attributes (i.e., skills for implementing the program), and individual identification with the organization. Lastly, the “Intervention Characteristics” questions will assess relative advantage, trialability, complexity, and design quality and packaging.

The CBO/FBO instruments (Table 2) will explore 5 domains: background/organizational structure, readiness and capacity, organizational workflow, partnership evaluation, and experiences of stress, racism, and racial discrimination. The questions in the “Background/Organizational Structure” domain will assess organizational characteristics and existing structures. The questions in the “Readiness and Capacity” domain will assess organizational preparedness to establish a referral partnership with primary care practices. “Organizational Workflow” questions will assess referral and partnership workflows at each site. “Partnership Evaluation” will assess organizational attitudes and beliefs about forming partnerships with outside organizations, and “Experience of Stress, Racism, and Racial Discrimination” will capture these experiences within the community and congregations.

Lastly, the NYULH Leadership and Provider interview questions (Table 2) will include 4 domains: Inner setting, Outer Setting, Engagement, and Stress/Discrimination in Hypertension Management. “Inner Setting” questions will assess capacity to implement and administer hypertension management at respondents’ site and institution; “Outer Setting” questions will assess patient experiences with a hypertension management program; “Engagement” questions will evaluate implementation leaders’ engagement with nurses working on hypertension management; “Stress/Discrimination” questions will explore organizational-level experiences of racism/discrimination. Interview guides were further tailored according to interviewee; thus, not all interviewees will receive questions from all domains.

### Secondary data

Secondary data analyses of EHR data and NYC Community Health Surveys data will provide additional context at the community, health system, and patient levels.

#### Data sources 

Secondary data for the needs assessment are from 2 main sources: (1) New York City Community Health Survey datasets and (2) NYU Health system Epic EHR. All secondary datasets will be obtained and managed by the study analyst. The full CHS 2018 and 2019 datasets will be downloaded under a data use agreement, approved by the NYC Department of Health and Mental Hygiene (DOHMH). The full datasets will be provided to the study analyst by an authorized NYC DOHMH representative. Patient-level Epic data from individual primary care practices will be acquired by the Clinical Research Data Management Core (DataCore) group at NYULH and provided to the study analyst. To ensure that patient confidentiality is maintained, the patient data will be de-identified and stored in a secure database. Only the study analyst and other pre-specified study team members will have access to identifying information.

#### Measures

##### 2018–2019 NYC community health survey measures

A descriptive analysis of 2018–2019 New York City CHS data will be used to characterize the outer setting in which PACE will operate. Measures explored in the datasets will include sociodemographic, behavioral, and relevant clinical variables, stratified by race, ethnicity, and HTN diagnosis. Sociodemographic variables will include health insurance status, age, sex, body mass index (BMI), marital status, parental status, number of adults in the household, education level, employment status, NYC borough, poverty level, perceived level of neighbors’ willingness to help fellow neighbors, and neighborhood poverty. Clinical variables will include whether respondents checked their BP in the last 30 days, HTN diagnosis, HTN medication(s) status, and whether participants were diagnosed with diabetes, obesity, or asthma. Behavioral variables will include smoking status, drinking status, fruit/vegetable consumption, sugar-sweetened beverage consumption, and physical activity status. Analysis of these variables will provide an overview of the population characteristics and comorbidity burden of the population to be engaged during PACE implementation.

##### NYU health system epic EHR (patient data)

A descriptive analysis of 2018–2021 NYULH EHR data, collected from the Epic platform, will help to characterize HTN burden and risk factors among patients within NYULH primary care practices. Measures explored in the datasets will include sociodemographic and clinical factors. Sociodemographic variables include race, health insurance status, age, sex, and NYC borough for current patients within NYULH practices. Clinical variables include medications (i.e.: HTN classes and dosages), medical comorbidity (i.e., diagnosis of diabetes, obesity, stroke, and/or chronic kidney disease), Charlson Comorbidity score, social history (i.e., smoking and drinking status), and patients’ clinic visit history. In addition, HTN diagnosis and BP readings (2018 to 2021) will be extracted from the EHR to categorize the patients in order to explore their HTN risk levels. Patients will be categorized into four groups: (1) HTN diagnosis with controlled BP; (2) HTN diagnosis with uncontrolled BP; (3) no HTN diagnosis and at least 2 uncontrolled BP readings (1 week apart); and (4) no HTN diagnosis and at least 2 elevated BP readings (1 week apart).

##### Ethics and data storage protocol

The study protocol and activities were approved by the NYU Grossman School of Medicine Institutional Review Board on November 19, 2021. Informed consent will be obtained from all study participants. Quantitative data collected for the purposes of this study will be anonymized and stored in a secure database under the participant’s unique identifier. Identifying information will be available only to the study analyst and approved study staff. All transcribed qualitative data will be anonymized and stored in secure, password-protected files accessible only to study staff.

#### Main outcome

The primary outcome of this needs assessment is a context-specific tailored practice facilitation strategy to be implemented among 25 primary care practices and 2 pilot sites in NYC, in order to improve HTN control among Black patients.

### Data analyses

All statistical analyses will be conducted in R (R Foundation for Statistical Computing, Vienna, Austria) and qualitative analyses will be conducted in Dedoose qualitative analysis software. Findings from all data sources will be integrated and triangulated to generate themes, supplemented by quantitative findings. We will prioritize findings from the qualitative data, which by nature may provide more robust findings and richer understanding of the implementation context. The triangulation of these data sources will inform the PF strategy. Below, we describe in-depth the analyses for each data source.

#### Primary data

##### Primary data analyses

**Quantitative surveys conducted among health systems leadership, providers, and staff**

Quantitative analysis of the surveys will involve conducting descriptive and advanced statistical analyses; we will summarize continuous variables with means, standard deviations, medians, and ranges and will summarize categorical variables with frequency distributions. For continuous variables, we will display the distribution of response and will run ordered logistic regression. We will run chi-square tests for binomial categories. Descriptive analyses of the site and respondent characteristics will be conducted to characterize the implementation context. Site and respondent characteristics will be represented using descriptive statistics (mean, standard deviations, frequencies) to provide documentation and description of the practices, implementation components, and context. Findings will be compared across sites.

**Qualitative analysis**

Data from the interviews and focus groups sessions will be automatically transcribed in the WebEx platform (virtual sessions) or manually transcribed verbatim by study staff (in-person sessions). Analyses will be independently conducted in Dedoose qualitative analysis software platform by study team members. Analyses will follow an open thematic coding process, using a mix of inductive and deductive primary coding. An a priori codebook, which captures key study measures, will be developed, upon which coders will build in order to capture emerging codes. Throughout the analysis, coders will utilize practice member checking to make sure that we have adequately captured emerging themes and reflections. Qualitative sessions will be double coded by two coders, and inter-rater reliability will be evaluated. Coders will reconcile interviews until adequate reliability is achieved (Krippendorff’s alpha > .80). Should data reach saturation, qualitative sessions and analyses will be concluded.

#### Secondary data

##### Secondary data analysis

**CHS 2018–2019 data**

Using the CHS datasets, we will conduct descriptive statistical analyses to characterize the catchment population. We will summarize categorical variables (measures described above) and perform frequency distributions. Survey weights from 2018 to 2019 will be combined to estimate the population. Sociodemographic and behavioral variables will be stratified by the variables “Race,” “Ethnicity,” and “Hypertension Diagnosis” to explore the relationships between these variables. Two 2-way contingency tables will examine “Race” and “Hypertensive Diagnosis” by all other variables. Chi-square test for categorical variables and Kruskal-Wallis test for ordinal variables will be used to check the relationships between the variables. A three-way contingency table will be conducted to examine “Hypertensive Diagnosis” and “Race” by all other variables. The table will include proportions across the two main variables of interest (i.e., Hypertensive Diagnosis and Race). A logistic regression model will be conducted to examine the *P*-values for significance and to calculate odds ratios for “hypertensive diagnosis” as response variables by “Race” and other variables as predictor variables.

**Electronic Health Record data**

To evaluate the 2018–2021 NYULH EHR data, baseline characteristics and outcomes will be summarized descriptively and graphically. Continuous variables will be summarized with means, standard deviation, medians, and ranges. Categorical variables will be analyzed with frequency distributions. We will group patients to create variables as follows: group 1 will contain patients with diagnosis of HTN and who have a controlled BP reading (systolic blood pressure [SBP] <130 and diastolic blood pressure [DBP] <80) based on mean of the last two BP readings; group 2 will contain patients with a diagnosis of HTN who have an uncontrolled BP reading (SBP≥130 or DBP ≥80) based on the mean of the last two BP readings; group 3 will contain patients without a diagnosis of HTN who have at least 2 uncontrolled BP readings; and group 4 will contain patients without a diagnosis of HTN who have at least 2 elevated BP readings (120≤SBP≤129 and DBP ≤ 80). To examine the associations between the variables, the chi-square test for categorical variables and the Kruskal-Wallis test for ordinal or interval variables will be used. *P*-values of these tests which are less than or equal to the specified significance level will be used to examine associations between variables. Two 2-way contingency tables will examine Race by all other variables and Group by all other variables. A three-way contingency table will be conducted to examine Hypertensive diagnosis and Race by all other variables. The table will examine proportions across the two main variables of interest (Hypertension diagnosis and Race). Lastly, we will conduct a multinomial logistic regression model for our nominal outcome variable, group, with *P*-values and odds ratios to examine the association between categories of groups.

##### Triangulation and synthesis of findings

Triangulation will be used to integrate the multiple data sources, in order to improve the understanding of the context in which PACE will be implemented in primary care practices in NYC. We will use a concurrent triangulation approach [49] to examine instances of data congruence and or incongruence. This will also help to strengthen our interpretation and ensure that we have captured all potential participants at risk in the community (informed by the CHS data) and those already in the NYU healthcare system but undiagnosed and uncontrolled. The focus group and interview discussions will help to inform our understanding of the quantitative data and collaboratively work with the various stakeholders to identify facilitators to implementation and derive potential solutions to contextual challenges during PACE implementation. Following best practices for mixed methods research [50], we will construct a joint display [51] that integrates the data sources. Constructs to be integrated include community and neighborhood characteristics, patient-level determinants of HTN control, organizational capacity for change, availability of resources to support change, and attitudes toward EBIs. Should the data be divergent, we will assign higher credence to the qualitative data which offer richer explanation about participants’ attitudes and behaviors [50]. We will incorporate best practices in data visualization to transform these large bodies of disparate data into timely, digestible, and actionable insights that will inform the PF strategy.

## Discussion

This protocol outlines the multilevel and multicomponent strategies used to understand the population and the context in which PACE will be implemented whilst engaging key clinic and community-level stakeholders to improve HTN control among Black patients. We aimed to develop a clinic-community-based PF program using three multi-level EBIs (NCM, RBPM, and SDoH support) integrated as a community-clinical linkage model for improved HTN control in Black patients. Blacks in the USA experience a disproportionate burden of HTN and resultant cardiovascular-related outcomes, compared with Whites [2]. Although achieving HTN control for Blacks is crucial for reducing poor cardiovascular outcomes and narrowing the racial mortality gap, low BP control rates still persist for this group [2]. Sustainable and scalable multi-level models that are tailored to clinical settings and conducted in partnership with the surrounding community are needed to reduce HTN burden among Blacks, thus mitigating existing disparities.

To our knowledge, this program will be the first study to examine the integration of these multi-level EBIs into a collaborative community-clinic linkage model. However, whilst implementation of such models is necessary to address the complex barriers to HTN control, it is insufficient without a rigorously developed and applied implementation strategy informed by results from a needs assessment to tailor the model to its context. Additionally, to achieve population-level impact outside of the original study catchment, and improve the city-wide HTN disparities, the intervention must be scalable and adaptable to varying implementation contexts and populations. Conducting and applying this needs assessment will inform a rigorously developed, applied, and tested PF model, thus increasing the efficacy, scalability, and adaptability of the intervention. Ultimately, applying the needs assessment findings to develop a tailored PF model may lead to greater population-level impact on reducing HTN burden among Blacks [37, 38], and provide insight as to effective strategies for engaging key stakeholders for community-clinic linkage model underpinning the program.

### Limitations

Although we are using robust strategies for this needs assessment, as with any study, there are limitations. One potential limitation is difficulty recruiting for the surveys, interviews and focus groups. We anticipate recruiting sufficient participants to achieve theoretical saturation and gain meaningful insights. However, there is a chance we may be unable to meet planned recruitment numbers. Use of purposive sampling within identified social networks is expected to increase likelihood of participation, where the study is known within participants’ networks. In the event that we do not meet planned recruitment numbers, we may triangulate findings with relevant literature, supplement the data with additional analyses of secondary datasets, or undergo a second round of recruitment. Another limitation is that we do not use probabilistic sampling methods for this study, which may limit generalizability. However, we believe that use of purposive sampling of key informants will enable us to gain the richest information to characterize implementation context; in this way, generalizability to the health system is not a key problem. A third limitation is that EHR data used for patient-level analyses may contain a certain proportion of missing or inaccurate data. We will mitigate this by performing data quality checks and/or imputation (i.e., maximum likelihood estimation, Multiple Imputation) to ensure sufficiency. Lastly, both social desirability and acquiescence biases could influence responses, since participants of qualitative components may be interviewed by known colleagues. This may cause participants to respond in ways that they perceive as more positive or acquiescent. To mitigate this, questions will be framed neutrally to indicate no clear “correct” or “positive” answer, and probes will be administered when needed to draw out more detailed responses.

The strengths of this study include triangulation of qualitative and quantitative data, involvement of multilevel stakeholders, continuous engagement of community and faith-based stakeholders in their context, and use of frameworks to guide study design and analysis. These strengths help to address limitations and bolster the validity of the findings.

## Conclusion

We describe a comprehensive needs assessment to understand hypertension management experiences of Black patients with uncontrolled HTN in NYC by collaborating with multiple clinic and community stakeholders to identify available capacities and supports for hypertension control for this group. Integration of stakeholders’ priorities, perspectives, and practices with the PACE program will improve adoption, sustainability, and the potential for scale-up. Findings will inform the development of a tailored PF strategy for implementing PACE into primary care settings for hypertension control to ensure intervention uptake and sustainment.

## Data Availability

Data sharing is not applicable to this manuscript as it is a study protocol.

## Abbreviations

BMI: Body mass index
BP: Blood pressure
CAB: Community Advisory Board
CBO: Community-Based Organization
CBPR: Community-Based Participatory Research
CCLM: Community-clinic linkage model
CDS: Clinical Decision Support
CFIR: Consolidated Framework for Implementation Research
CHW: Community health worker
CHS: Community Health Survey
DataCore: Clinical Research Data Management Core
DBP: Diastolic blood pressure
DOHMH: Department of Health and Mental Hygiene
EBI: Evidence-based intervention
EHR: Electronic Health Record
FBO: Faith-based organization
HIPAA: Health Insurance Portability and Accountability Act
HTN: Hypertension
NCM: Nurse Case Management
NYC: New York City
NYU: New York University
NYULH: New York University Langone Health
PACE: Practice Support and Community Engagement
PF: Practice facilitation
RBPM: Remote blood pressure monitoring
REDCap: Research Electronic Data Capture
SBP: Systolic blood pressure
SDoH: Social determinants of health
